# The Indulgence of Grief

**Published:** 1893-08

**Authors:** Leander J. White


### THE INDULGENCE OF GRIEF.

It is not in the power of every one to prevent the calamities of life—but it evinces true magnanimity to bear up under them with fortitude and serenity. The indulgence of grief is made a merit of by many, who, when misfortunes occur, obstinately refuse all consolation, till the mind, oppressed with melancholy, sinks under its weight. Such conduct is not only destructive to health, but inconsistent with reason, religion and common sense. "There are what may be called the ceremonies of sorrow; the pomp and ostentation of effeminate grief, which speak not so much the greatness of the misery as the smallness of the mind."

"To persevere
"In obstinate condolement, is a course
Of impious stubborness, unmanly grief,
It shows a will most incorrect to Heaven,
A heart unfortified, a mind impatient;
An understanding simple and unschooled."

Change of ideas is as necessary to health as change of fortune. When the mind dwells long upon one subject — especially if it be of a disagreeable and depressing nature, it injures all the functions of the body. Hence the prolonged indulgence of grief spoils the digestion and destroys the appetite. The spirits become habitually depressed—the body emaciated and the fluids deprived of their appropriate supply of nutriment from without, are greatly vitiated. Thus, many a constitution has been seriously injured by a family misfortune, or any occurrence giving rise to excessive grief.

It is, indeed, utterly impossible that any person of a dejected mind should have good health.

Life may, it is true, be dragged on for years. But whoever would live to good old age, and vigorous withal, must be good humored and cheerful. This, however, is not at all times in our power—yet our temper of mind, as well as our actions, depends greatly on ourselves.

We can either associate with cheerful or melancholy companions—mingle in the offices and amusements of life—or sit still, and brood over our calamities, as we choose.

These and many similar things, are certainly within our power, and from these the mind very commonly takes its complexion.

The variety of scenes which present themselves to our senses, were certainly designed to prevent our attention from being too constantly fixed upon one single object.

Nature abounds with variety, and the mind, unless chained down by habit, delights in the contemplation of new objects.

Few persons are hurt by grief, if they pursue their business or their active duties with attention. But it is to be lamented that too many persons, when overwhelmed with grief, betake themselves to the intoxicating bowl. This is making the cure worse than the disease, and seldom fails to end in the ruin of fortune, character, happiness and constitution. LEANDER J. WHITE.

